# Estimated future incidence of malignant mesothelioma in South Korea: Projection from 2014 to 2033

**DOI:** 10.1371/journal.pone.0183404

**Published:** 2017-08-17

**Authors:** Kyeong Min Kwak, Domyung Paek, Seung-sik Hwang, Young-Su Ju

**Affiliations:** 1 Department of Environmental Health Sciences, Graduate School of Public Health, Seoul National University, Seoul, Republic of Korea; 2 Department of Occupational and Environmental Medicine, Gachon University Gil Medical Center, Incheon, Republic of Korea; 3 Department of Public Health Science, Graduate School of Public Health, Seoul National University, Seoul, Republic of Korea; 4 Department of Occupational and Environmental Medicine, Hallym University Sacred Heart Hospital, Anyang, Republic of Korea; University of Montana, UNITED STATES

## Abstract

Malignant mesothelioma is a malignant tumor on the pleura or the peritoneum caused mostly by asbestos. Although asbestos is not currently used in South Korea, the incidence of mesothelioma is increasing due to its long latent period. This study predicted the incidence of malignant mesothelioma in South Korea over the next 20 years using an age-period-cohort (APC) model. Data regarding mesothelioma incidence from 1994–2013 were acquired from the Korea Central Cancer Registry (KCCR). Demographic data, including prospective resident data, were acquired from the Korean Statistical Information Service (KOSIS) for 1994–2033. An APC model with Møller’s power-link function was utilized to predict the incidence of mesothelioma. It was predicted that 2,380 and 1,199 new cases of mesothelioma in men and women, respectively, would occur over the next 20 years. For both sexes, the mesothelioma incidence rate was predicted to be greater in 2029–2033 compared to that in 2009–2013 (men, 0.282 vs 0.563; women, 0.155 vs 0.217). For men, the age-standardized incidence rate was predicted to be slightly greater in 2029–2033 relative to the rate in 2009–2013 (0.228 vs 0.235), while the age-standardized incidence rate in women decreased within the same timeframe (0.113 vs 0.109). The changes in mesothelioma incidence were mostly caused by changes in the population structure due to aging and not by changes in the mesothelioma risk ratio. The results of this study project a continuous increase in mesothelioma incidence in South Korea over the next 20 years. Although the projected increase in mesothelioma incidence was not related to an increase in the mesothelioma risk ratio, continuous preventive efforts are necessary to reduce the exposure to asbestos and prevent the trend from worsening.

## Introduction

Malignant mesothelioma is a malignant tumor on the pleura or the peritoneum. Asbestos causes the majority of malignant mesothelioma with an attributable risk of 80–90% [1]. The prognosis is poor with a median survival time of 12 months after diagnosis [2].

In South Korea, the use of asbestos began in the 1930s with the development of asbestos mines. Although the use of asbestos temporarily decreased following independence, economic growth in the 1970s called for the importation of large amounts of asbestos for use in construction materials, machinery parts, and insulators [3]. Asbestos use peaked in the early and mid-1990s. The use of toxic crocidolite and amosite was banned in 1997 and asbestos imports sharply declined [4]. In 2007, the use of asbestos in construction materials was banned, and the import/use of all types of asbestos was officially banned in 2009. Despite the ban on asbestos use, asbestos-containing construction materials still expose construction workers and civilians to asbestos, especially during demolition or reconstruction processes [5]. Unlike lung cancer, malignant mesothelioma can be caused by exposure to small amounts of asbestos [6] and has a long latent period of 20–40 years [7]. Therefore, the incidence of mesothelioma is still increasing, despite the ban on asbestos.

Malignant mesothelioma caused by asbestos is an important topic in other countries as well. In Australia, 30 out of every million individuals develop malignant mesothelioma, while in many European countries, including Great Britain, 10–30 out of every million individuals develop the malignancy [8]. In the USA, nine out of every million individuals develop malignant mesothelioma, while in Japan seven out of every million individuals develop the malignancy [8]. In the Western society, malignant mesothelioma is regarded as a disease that is expected to continuously increase, surpassing the past incidence, and will cost an estimated 300 billion dollars in the next 20 to 30 years [9]. Moreover, Japan [10], Great Britain [11] the Netherlands [12], Canada [13], and Italy [14] have conducted studies predicting the future incidence of malignant mesothelioma. Using the reported temporal correlation between peak asbestos use and the incidence of malignant mesothelioma determined in these studies, the Ministry of Environment (ME) of South Korea has predicted that the number of domestic malignant mesothelioma cases would peak in 2045 [15]. However, unlike other countries, no quantitative studies regarding the predicted incidence of malignant mesothelioma have been conducted in South Korea.

The current incidence of malignant mesothelioma in Korea is lower than that in the US, Europe, and Japan, but is gradually increasing [16]. Since the introduction and wide use of asbestos occurred later in South Korea than in other countries [17], the prediction of the future incidence of mesothelioma has important implications from a public and environmental health standpoint. Therefore, we aimed to determine the incidence of malignant mesothelioma over the next 20 years (2014 to 2033) in South Korea using an age-period-cohort (APC) model based on statistics from the Korean Central Cancer Registry (KCCR).

## Materials and methods

### Data

Data on mesothelioma (C45) incidence from the last 20 years (1994 to 2013) were obtained from the KCCR. The Korean government installed the KCCR in 1980 and promoted the registration of cancer cases. Data from the Registry are highly accurate for the diagnosis of cancer and are considered reliable in determining the actual incidence [18, 19]. The incidence of malignant mesothelioma was organized according to sex in 5-year epochs (1994–1998, 1999–2003, 2004–2008, and 2009–2013). In order to determine the incidence rate, the annual resident registration data (1994–2013) and prospective resident data (2014–2033) were obtained from the Korean Statistical Information Service (KOSIS). Resident data were also organized in 5-year epochs (1994–1998, 1999–2003, 2004–2008, 2009–2013, 2014–2018, 2019–2023, 2024–2028, 2029–2033) (S1 Table). Mesothelioma incidence and resident data were further divided into age groups using 5-year units (0–4, 5–9, 10–14, 15–19, 20–24, 25–29, 30–34, 35–39, 40–44, 45–49, 50–54, 55–59, 60–64, 65–69, 70–74, 75–79, 80–84, 85+ years of age). Age-standardized incidence rates (ASRs) were investigated using world-standardized population data (S2 Table) from the World Health Organization (WHO) [20].

### Statistical modeling

An APC model was used to estimate the prospective incidence rate of malignant mesothelioma. APC modeling is a widely used method to predict cancer occurrence. There are three components to the method: age (at the time of disease onset), period (the year of disease onset), and cohort (the birth date of the affected person). In cancer cases, age is assumed to play a bigger role relative to the period and cohort, which often leads to the negligence of the period and cohort. Short-term cancer onset estimations (5 years or less) are usually performed using an age-period model, while long-term estimations are typically based on APC models [21].

The APC model uses a generalized linear model framework and several other link functions, which are taken into account in order to predict the average value. The most representative models are the Poisson regression model with a log-link function and the power-link model. For example, Osmond et al. estimated the mortality of lung cancer using a Poisson regression model with a log-link function [22]. However, in Møller et al., the authors recommended using a power-link function instead of the log-link function [23]. Møller's study has shown that models using a power-link function with a power of 5 produced predictions similar to the actual incidence. Moreover, the former model assumes that the predicted value reflects an exponential growth, which has been criticized for the possibility of drastic predictions [24, 25]. The model using a power-link function is fortified against this problem [21]. Møller et al. used an APC model based on a power-link function to predict the cancer incidence in Nordic nations and Great Britain [26, 27]. The model is shown below:

$${\text{R}}_{\text{ap}}={\left({\text{A}}_{\text{a}}+\text{D}\cdot \text{p}+{\text{P}}_{\text{p}}+{\text{C}}_{\text{c}}\right)}^{5}$$

R_ap_: The incidence for a specific age range (a) during a specific period (p)

A_a_: The age component of a specific age range (a)

D: Common drift parameter reflecting the linear component of the trend

P_p_: The nonlinear period component of a specific period (p)

C_c_: The nonlinear cohort component in a specific cohort (c)

This model takes into account the drift parameter, as well as the age, period, and cohort components, in determining the average trend. As the effects of the current data decrease with time, the drift parameter was continuously decreased by 25% every 5 years. In addition, when the observed incidence showed any significantly sharp curvature, only the data from the last 10 years was used in the projection. When the observed incidence did not show any significantly sharp curvature, the average trend of the entire observation period was used for projection. In this study, the lowest age was set to 25 years. The possible incidence in cases with patients below the age of 25 years was calculated using the average incidence for the last 10 years in the projection.

In this study, the crude incidence rate and ASR for malignant mesothelioma from 2014 to 2033 were estimated for both sexes using Møller’s model. The Nordpred package, which was developed by the Norwegian cancer registry based on Møller’s APC model, was used for statistical analysis. Nordpred software is based on R, which can be downloaded from the internet [28, 29].

### Ethics statement

This study was conducted after obtaining approval from the IRB of Hallym University Sacred Heart Hospital (IRB No. HUSHHIRB-2016-I116).

## Results

Over the last 20 years (1994–2013), among an average annual Korean population of 48,460,000, mesothelioma occurred in 946 men and 534 women. In the next 20 years, it was estimated that 2,380 and 1,199 new cases of mesothelioma will occur in men and women, respectively. In men, the number of mesothelioma cases was predicted to increase until 2029–2033. However, in women, the incidence of mesothelioma was predicted to increase until 2024–2028 and decrease until 2029–2033 (Table 1).

**Table 1:** Observed (1994–2013) and predicted mesothelioma cases (2029–2033) in South Korea, stratified according to sex.

	1994–1998	1999–2003	2004–2008	2009–2013	2014–2018	2019–2023	2024–2028	2029–2033
**Men**	138	175	276	357	452	551	648	729
**Women**	79	100	161	194	272	317	325	285

**Fig 1:**
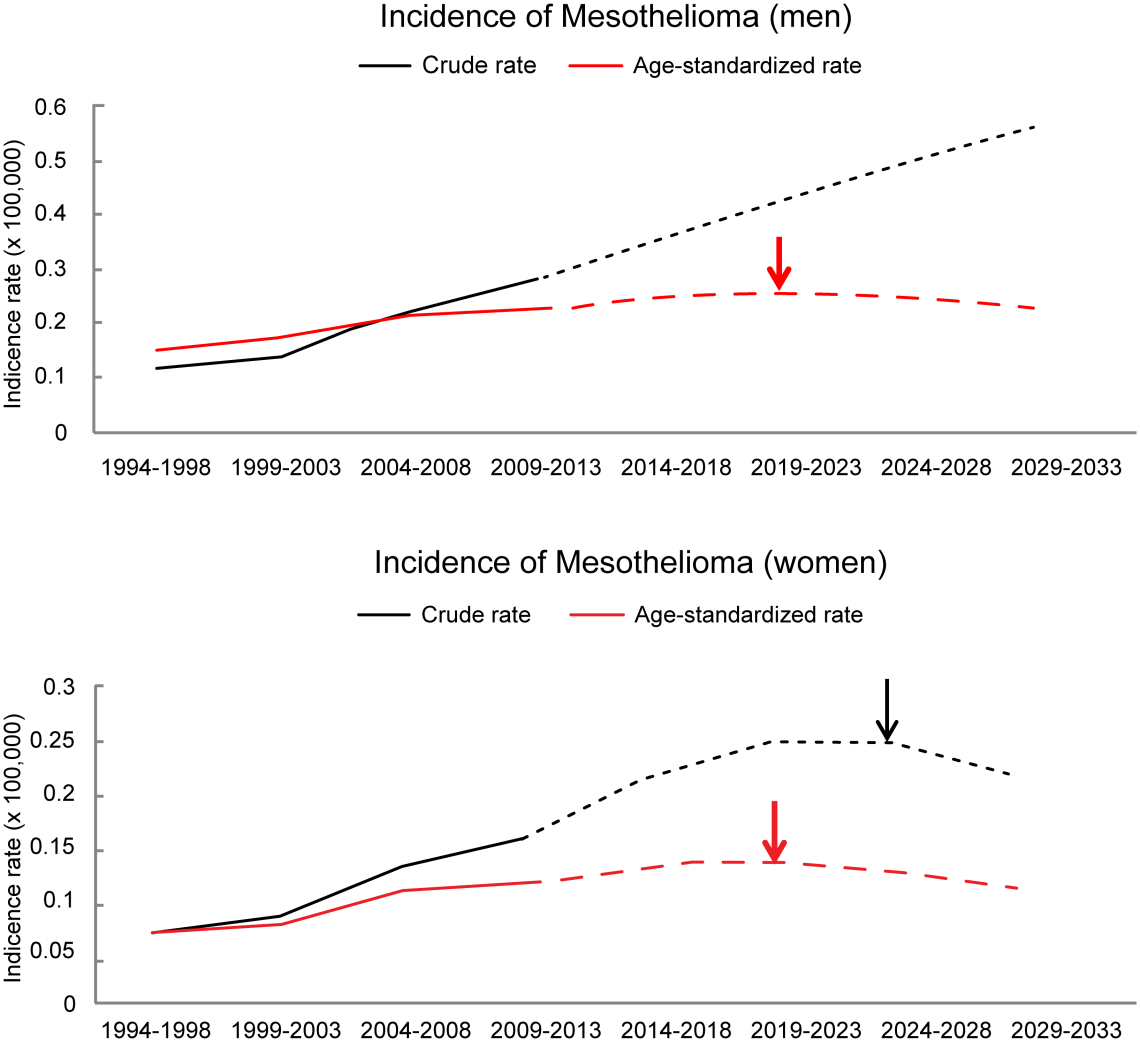
The crude and age-standardized incidence rate (per 100,000) for mesothelioma in South Korea during the observed (1994–2013) and predicted periods (2014–2033), stratified according to sex. The arrows indicate the peak incidence rate for mesothelioma. (The peak crude incidence rate does not appear by 2033 in men).

In both sexes, the crude incidence rate was predicted to be higher in 2029–2033 than in 2009–2013 (men, 0.282 vs 0.563; women, 0.155 vs 0.217). However, the trends were different for men and women. In men, the expected crude incidence rate was estimated to increase continuously until 2029–2033. In women, the expected crude incidence rate continuously increased until 2024–2028 and decreased afterwards. In men, the expected ASR for mesothelioma was slightly higher in 2029–2033 compared to that for 2009–2013 (0.228 vs 0.235). In women, the ASR was slightly lower in 2029–2033 compared to that for 2009–2013 (0.113 vs 0.109). However, the changes were very small. The trend in ASR showed a continuous and slow increase until 2019–2023 for both sexes, and slowly decreased afterwards (Fig 1).

The overall changes in the incidence of mesothelioma can be related to changes in the risk ratio for mesothelioma and/or changes in the population size and age distribution. Therefore, the ASR of mesothelioma was defined as the pure risk ratio for mesothelioma that was not related to population size and structure. Compared with the current period (2009–2013) and that for 20 years from now (2029–2033), the number of incidence cases was estimated to increase 104.2% (357 to 729) and 46.9% (194 to 285) in men and women, respectively. Changes in the number of mesothelioma incidence cases due to changes in mesothelioma risk ratio were very small (men, 2.7%; women, -2.9%). The increase in the number of mesothelioma incidence cases was mostly due to changes in the population (men, 101.5%; women, 49.8%), particularly with changes in age distribution (men, 99.3%; women, 44.7%) (Table 2).

**Table 2:** The observed and predicted mesothelioma incidence between 2009–2013 and 2029–2033 in South Korea, stratified according to sex, with the corresponding percentage change in the number of cases decomposed into changing risk and demographic components.

	2009–2013		2029–2033				
	No. of cases	ASR	No. of cases	ASR	Change overall (%)	Change due to	
						Risk (%)	Population (%) (age structure, population size)
**Men**	357	0.228	729	0.235	104.2	2.7	101.5 (99.3, 2.2)
**Women**	194	0.113	285	0.109	46.9	-2.9	49.8 (44.7, 5.1)

**Fig 2:**
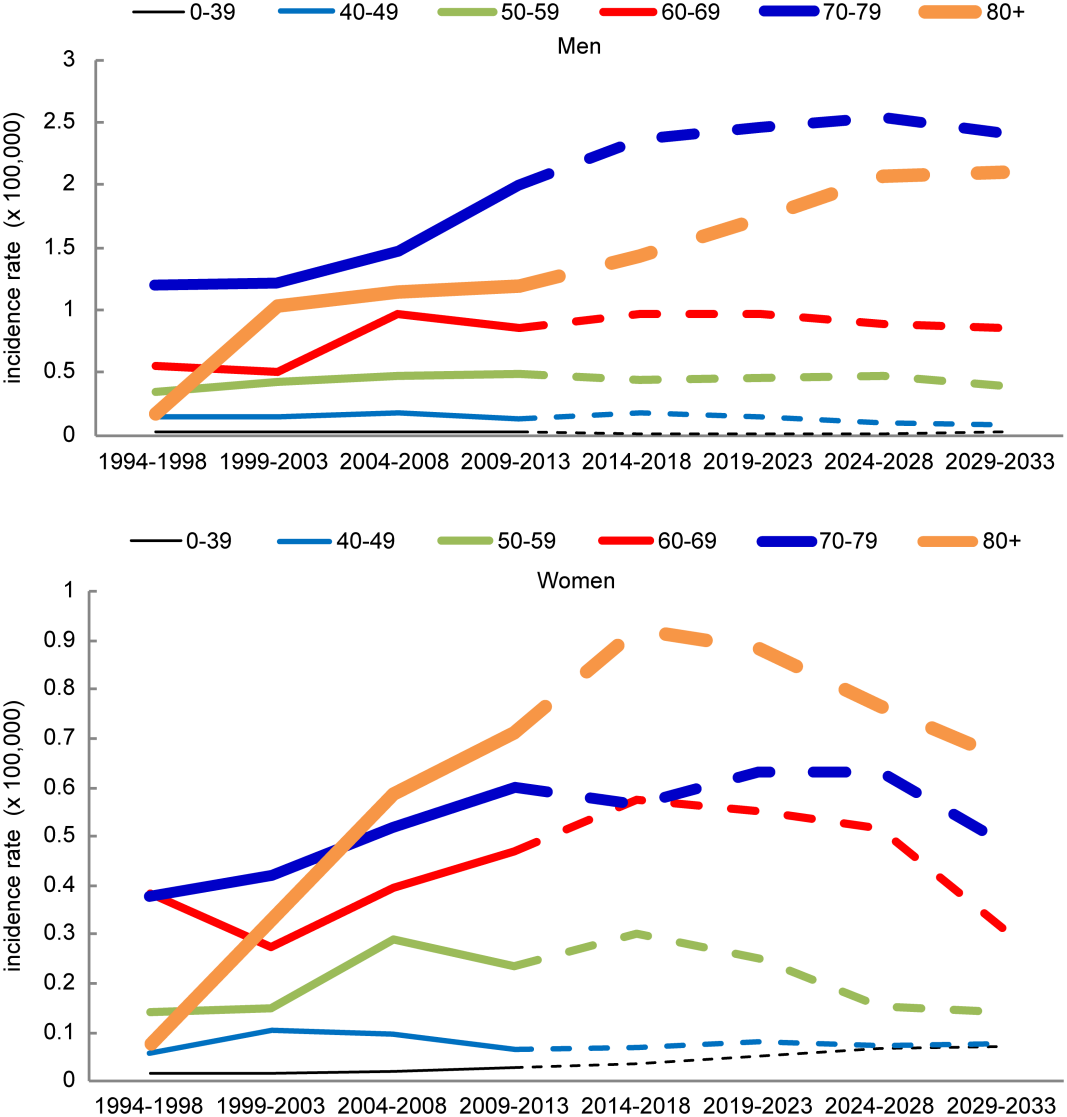
The age-specific incidence rates of mesothelioma in South Korea for the observed (1994–2013) and predicted periods (2014–2033), stratified according to sex.

**Fig 3:**
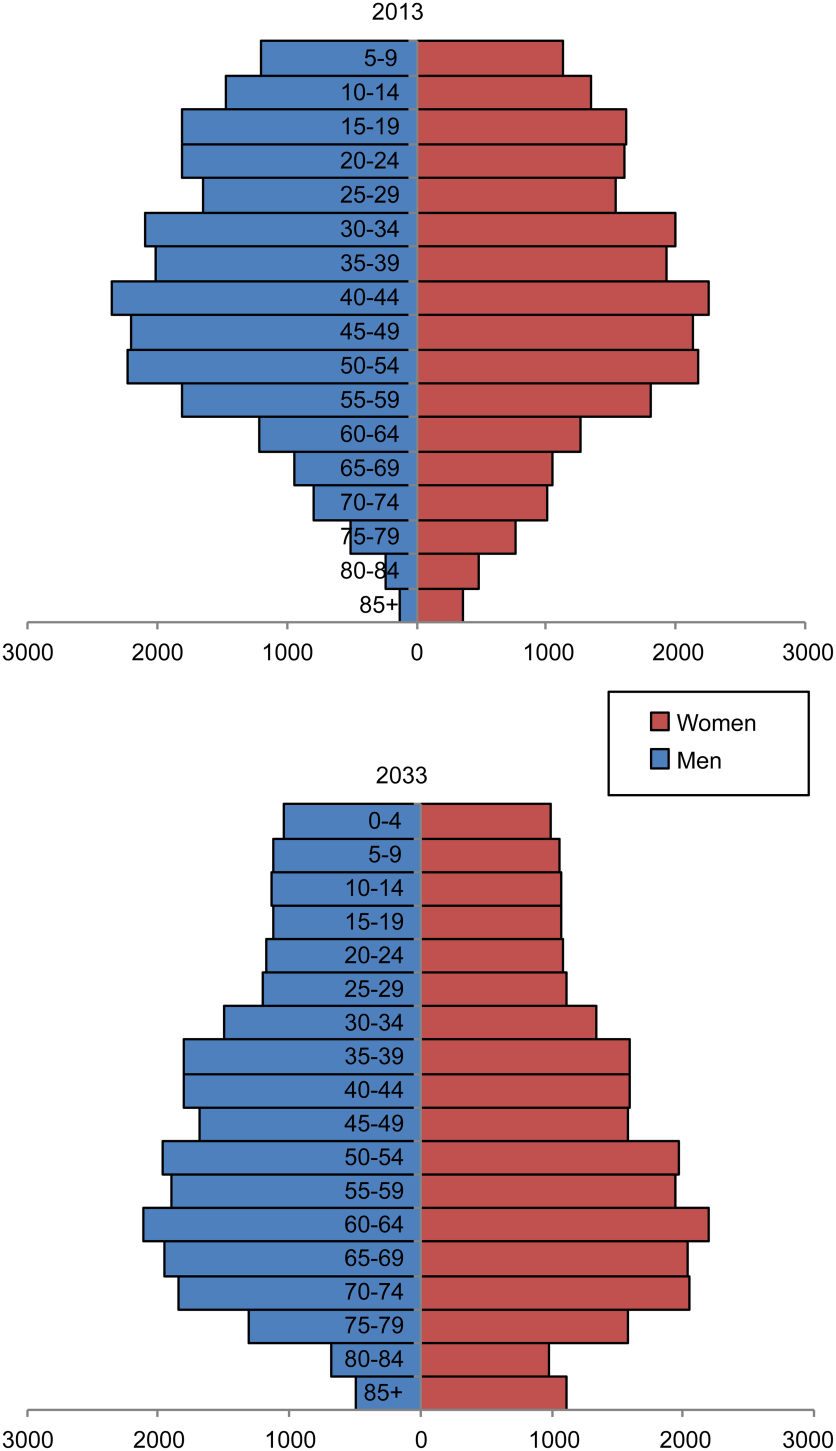
The population pyramid, stratified according to sex, for South Korea in 2013 and 2033.

The age-specific mesothelioma incidence was highest in high-age groups. In men, the incidence was the highest for those in their 70s while in women, the incidence was the highest for those in their 80s. In men, it was expected that the age-specific incidence would initially increase until it reaches a stationary phase. In women, the age-specific incidence initially increased in age groups above 50 years of age, and started to decrease after a certain point (starting in 2014–2018 for the following age groups: 50–59, 60–69, and 80+ years of age; and starting in 2024–2028 for those with 70–79 years of age). The incidence of mesothelioma was predicted to drastically increase in age groups above 60 years of age compared to those below 60 years of age, peaking for men in their 70s and for women in their 80s (Fig 2). In 2013, there were 3,814,000 males and 4,924,000 females who were above 60 years old in South Korea. In 20 years (2033), it was predicted that there would be 8,393,000 men and 9,941,000 women above the age of 60 years, reflecting an increase of 120% and 102% for men and women, respectively. In 2013, there were 1,654,000 men and 2,611,000 women who were above the age of 70 years. In 2033, it was predicted that there would be 4,328,000 men and 5,710,000 women above the age of 70 years, reflecting an increase of 162% and 119% for men and women, respectively (Fig 3). Therefore, the increase in the crude incidence rate of mesothelioma is mostly caused by changes in population structure due to aging rather than changes in the mesothelioma risk ratio.

**Fig 4:**
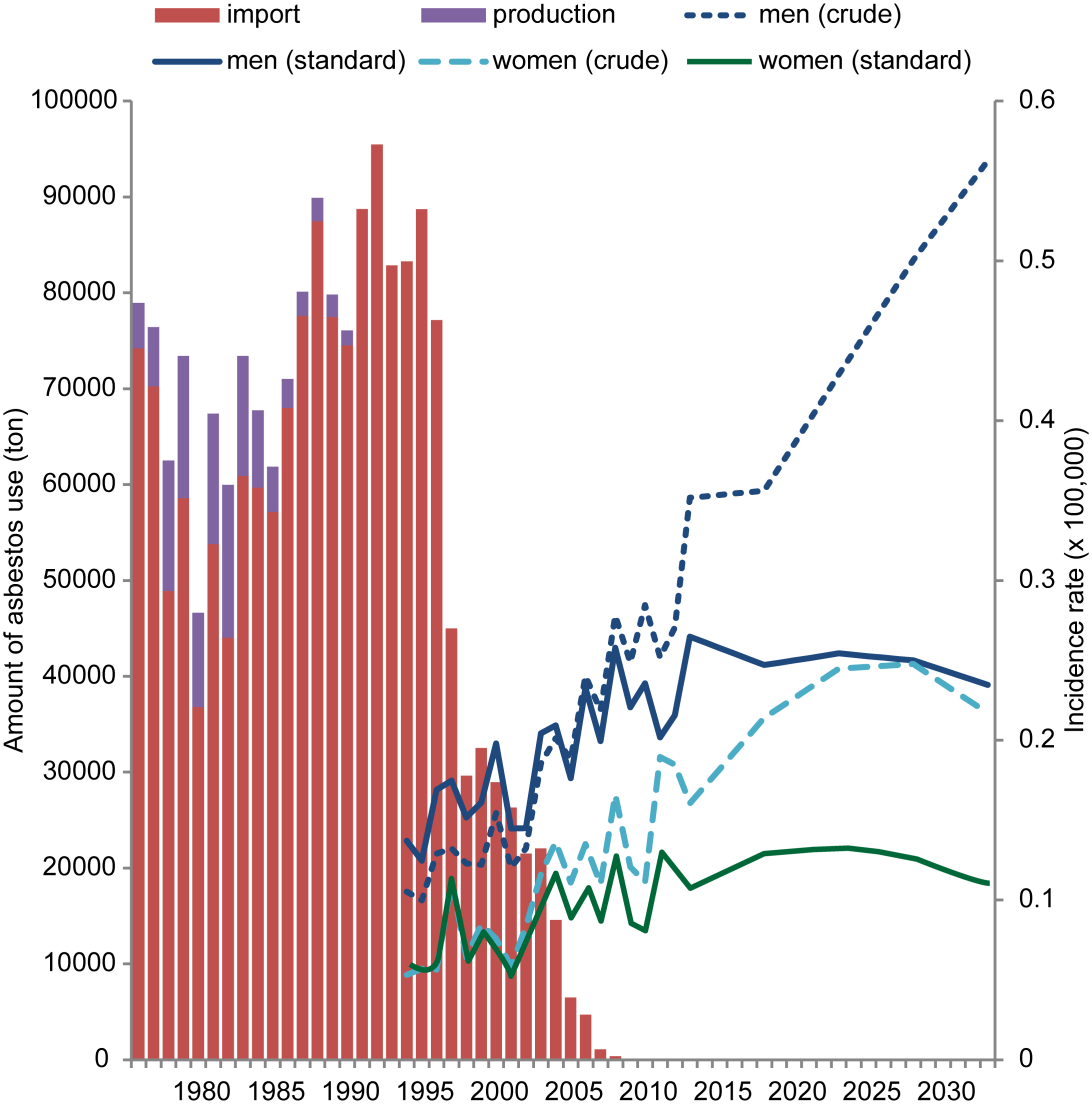
The amount of imported and produced asbestos (1975–2008) plotted against the age-standardized and crude incidence rates in South Korea during the observed (1994–2013) and predicted periods (2014–2033). (The observed incidence rates reflect annual data).

Although asbestos use was not utilized in the projection, changes in asbestos use over time and mesothelioma incidence were visually compared, as asbestos is a major cause of malignant mesothelioma (Fig 4). The export of asbestos was negligible (0 to 157 tons per year) and the production of asbestos was approximately 10,000 tons a year between the late 1970s and the mid-1980s. However, asbestos production was close to none after entering the 1990s due to financial reasons; thus, asbestos use depended solely on imports. The amount of asbestos imported peaked in the mid-1990s. Given that the mesothelioma ASR in both men and women peaked in 2019–2023, there is a gap of 25–30 years between peak asbestos use and peak ASR. This gap period is similar in duration to the latent period of mesothelioma, indicating that an increase in asbestos use is related to the increase in risk for mesothelioma after the latent period.

## Discussion

Malignant mesothelioma is very rare in South Korea, with a crude incidence rate of 0.26 per 100,000 in 2013 [30]. However, the rate is expected to increase. In this study, different trends were observed for men and women. The crude incidence rate was projected to increase continuously until 2029–2033 in men, and to increase continuously until 2024–2028, decreasing afterwards in women. For both sexes, the ASR was projected to increase continuously and slowly until 2019–2023, decreasing afterwards. The number of mesothelioma incidence cases is expected to increase by 104.2% and 46.9% for men and women, respectively, in 2029–2033 compared to that for the present (2009–2013). These findings were determined to be mostly caused by changes in population size and age distribution, rather than by changes in mesothelioma risk.

The present study was the first to estimate the future incidence of malignant mesothelioma in South Korea. Europe, United States, Canada, and other countries have predicted the incidence of mesothelioma using different estimation models. Krupoves et al. estimated the incidence of malignant mesothelioma in Canada using an age-cohort model, which indicated that the age-standardized incidence rate would peak between 2003 and 2012 in men, and between 2008 and 2017 in women, decreasing afterwards [13]. Murayama et al. estimated the mortality rate of pleural malignant mesothelioma in Japan using an age-cohort model, which indicated that the mortality rate would peak between 2030 and 2034 [10]. Hodgson et al. estimated the mortality rate of malignant mesothelioma in Great Britain using a Poisson regression model, which indicated that the mortality rate would peak between 2011 and 2015 [11]. Segura et al. estimated the number of deaths from pleural mesothelioma in the Netherlands using an age-cohort model, which indicated that the number of deaths would peak in 2017, and decrease afterwards. Given that the studies from Japan, Great Britain and the Netherlands estimated the mortality rate or number of deaths, rather than the incidence rate, and did not standardize for age, it cannot be determined whether the changes in mortality rate were caused by changes in the actual mesothelioma risk or by changes in population size and age distribution. A study in the United States estimated the incidence of mesothelioma using an age-cohort model; as the changes in the age-adjusted rate between 1990 and 2000 were flat, the projection showed that the incidence rate would peak between 2000 and 2004 [31]. In South Korea, Jung et al. estimated the incidence and mortality rates for major cancers in 2016 using a joinpoint regression model [32]. In addition, Son et al. estimated the mortality rate for Korean major cancers in 2032 using a Møller’s APC model similar to that in the present study [33]. However, mesothelioma was not examined in the two studies conducted in South Korea.

Several countries have used different models to predict the incidence of mesothelioma. In an age-cohort model, the period effect is not considered. The APC model is more stable than the age-cohort model and is more often utilized in estimating cancer incidence [34]. Poisson regression can be used to estimate the incidence of malignant mesothelioma by creating a regression model that includes total asbestos exposure and the age-specific exposure potential. This model also considers the lag period between asbestos exposure and disease, as well as the half-life of asbestos clearance from the lung. However, it would be difficult to use this as a prediction model with the present data, as setting the age-specific asbestos potential, lag period, and asbestos clearance would be arbitrary. Joinpoint regression is used to estimate changes in trends [35]. The annual rate of change is estimated by fitting several linear regression models that best describe the trends. This model can be used for short-term or single-year forecasts, but not for long-term forecasts. For these reasons, we chose an APC model and used Møller’s function based on this model for prediction.

In the present study, the sex ratio of malignant mesothelioma in South Korea was observed to be approximately 1.9:1, with male predominance. Other countries have reported sex ratios of 3:1 or 4:1 [36]. The higher ratio of women with malignant mesothelioma in South Korea is likely due to the relatively high proportion of cases exposed to environmental asbestos [37]. Although men are more often exposed to occupational asbestos relative to women, women are exposed to environmental asbestos to the same extent as men [38]. There are many old asbestos mines in the Chungcheong provinces. Since the mines have been abandoned and are not properly managed, residents in the surrounding area have been exposed to asbestos. Research regarding the incidence of malignant mesothelioma in residents living near the asbestos mines has not yet been conducted. However, the 2008 epidemiological survey of the ME found that a large number of asbestosis patients lived within 2 km of the asbestos mines in the Chungcheong province [5, 39]. The results of this survey confirm that environmental asbestos exposure is high in the residents near the asbestos mines. In addition, Korea rapidly became industrialized and urbanized, with many large-scale demolition works. This demolition exposed the residents in the surrounding area to environmental asbestos. Another source of environmental exposure concerns asbestos textile factories, which were widely used in the 1970s and 1980s [37]. Most workers in this industry are women and are likely exposed to asbestos.

Although comparing the incidence of pleural mesothelioma and peritoneal mesothelioma is important, as peritoneal mesothelioma is less associated with asbestos [40], the KCCR did not distinguish between pleural mesothelioma and peritoneal mesothelioma. In addition to the KCCR, malignant mesothelioma occurrence has been recorded in the mesothelioma surveillance system since 2001. Although this surveillance system involves passive reporting, resulting in an underestimation of cases, the database can provide a rough estimate of the proportions of pleural and peritoneal mesothelioma in South Korea. Among the 399 cases of malignant mesothelioma reported to the surveillance system between 2001 and 2010, 267 cases (66.9%) involved pleural mesothelioma, and 108 cases (27.1%) involved peritoneal mesothelioma [37]. Thus, peritoneal mesothelioma comprises more than 1/4 of all cases, and other factors besides asbestos may have affected the occurrence of mesothelioma.

Besides asbestos, erionite, a natural mineral fiber, constitutes an environmental exposure that causes malignant mesothelioma [41]. However, there is no known erionite in South Korea. In Yeongil, the Gyeongbuk province, etc., zeolite is present, but it does not contain erionite [42, 43]. Zeolite with the exception of erionite is not carcinogenic (IARC group 3 classification). Therefore, mineral fibers such as erionite might not be a cause of malignant mesothelioma in South Korea.

In addition, SV40 in contaminated polio vaccines [44], and germline BAP1 mutations [45] have been associated with malignant mesothelioma. The results from studies on SV40 are inconsistent and differ by geographical location [46]; however, recent studies suggest that a more widespread exposure to SV40 exists [47]. In South Korea, a study utilizing data from the malignant mesothelioma surveillance system failed to confirm that SV40 was associated with the development of mesothelioma [48]. However, the SV40 test was performed in only some of the patients; thus, the results of this previous study alone cannot be said to indicate that SV40 is unassociated with malignant mesothelioma in South Korea. Germline BAP1 mutations also can increase the incidence of mesothelioma [49]; however, the BAP1 test is not generally performed, and studies on BAP1 mutations have not yet been conducted in South Korea. Since SV40 and BAP1 tests are uncommon in South Korea, the possibility of malignant mesothelioma due to these factors cannot be excluded. Further studies are needed regarding the potential associations between malignant mesothelioma and SV40 exposure and BAP1 mutations.

There are several limitations in the present study. First, the APC model used in this study estimated the incidence rate of mesothelioma assuming that the past trend would be reflected in the future trend. Unpredictable occurrences in the future were not taken into account; therefore, the results could differ from the actual incidence of mesothelioma. In addition, the present study used mesothelioma incidence data obtained from the KCCR. Although the registry began in 1980, the registry was initially based on hospital records and the reliability was low. Population-based regional cancer registry (PB-RCR) programs subsequently started in 1991 in different regions [19]. Therefore, since the early cancer registry provided data with low reliability and additional data could not be easily gathered, only cancer data from the last 20 years were used for the projection. Furthermore, malignant mesothelioma is a very rare cancer and is not easy to diagnose; thus, controversy exists regarding the accuracy of the diagnosis. For example, a considerable number of cases diagnosed with malignant mesothelioma in China (43%) and France (33%) could not be verified [50, 51]. A panel of immunohistochemical stains is used to diagnose almost all recent cases of mesothelioma [52], and the present reliability of the diagnosis of mesothelioma in South Korea is considered high. However, in earlier cases of malignant mesothelioma, the presence of misdiagnoses may be non-negligible, as a panel of immunohistochemial stains was not necessary in many cases. Moreover, there is currently no validation study on the reliability of the diagnosis of malignant mesothelioma in South Korea. Thus, there may be a considerable number of misdiagnosed cases in earlier diagnoses of malignant mesothelioma in South Korea, similar to that in other countries. These potential misdiagnoses may act as a confounder in our projection. Finally, the amount of asbestos use was not considered in the estimation model; therefore, the effects of the amount of asbestos use on mesothelioma risk could not be identified.

The results of this study indicate that the ASR for malignant mesothelioma will slowly increase, with a peak in 2019–2023 and decreasing afterwards. However, for men, the actual number of incidence cases and crude incidence rate were predicted to further increase until 2033, while those in women were projected to increase until 2024–2028. Although asbestos is no longer used in South Korea, the results of this study project a continuous increase in mesothelioma incidence. Therefore, exposure to asbestos should be avoided to prevent worsening of the projected trend. Further studies should be conducted on the projected incidence rates of other diseases caused by asbestos, such as asbestosis.

## Supporting information

S1 Table 5-year resident registration (1994–2013) and prospective resident data (2014–2033). (DOCX)

S2 Table WHO world standard population (2000–2025). (DOCX)
