# Supplementary material for: Estimated future incidence of malignant mesothelioma in South Korea: Projection from 2014 to 2033
Source: PLoS One. 2017 Aug 17;12(8):e0183404. doi: 10.1371/journal.pone.0183404 (PMC5560642; doi:10.1371/journal.pone.0183404)
Supplement: S1 Table — (DOCX) [file pone.0183404.s001.docx]

**S1 Table. 5-year resident registration (1994-2013) and prospective resident data (2014-2033)**

| **Age Group** | **1994-1998** | **1999-2003** | **2004-2008** | **2009-2013** | **2014-2018** | **2019-2023** | **2024-2028** | **2029-2033** |
| --- | --- | --- | --- | --- | --- | --- | --- | --- |
| **Men** |  |  |  |  |  |  |  |  |
| 0-4 | 8,195,107 | 7,310,954 | 5,762,186 | 5,584,641 | 5,513,460 | 5,472,924 | 5,400,178 | 5,116,327 |
| 5-9 | 7,715,675 | 8,258,275 | 7,391,714 | 5,837,470 | 5,535,031 | 5,475,255 | 5,432,576 | 5,358,423 |
| 10-14 | 8,557,108 | 7,692,086 | 8,233,613 | 7,392,873 | 5,770,130 | 5,491,262 | 5,428,383 | 5,382,837 |
| 15-19 | 9,788,987 | 8,504,561 | 7,657,467 | 8,230,751 | 7,261,200 | 5,736,823 | 5,454,823 | 5,388,520 |
| 20-24 | 10,410,454 | 9,732,932 | 8,470,468 | 7,678,838 | 8,110,515 | 7,303,373 | 5,769,386 | 5,483,833 |
| 25-29 | 11,258,964 | 10,379,373 | 9,709,348 | 8,546,025 | 7,887,894 | 8,231,257 | 7,411,512 | 5,854,440 |
| 30-34 | 10,613,744 | 11,179,802 | 10,321,438 | 9,789,462 | 8,736,594 | 7,839,427 | 8,177,030 | 7,359,723 |
| 35-39 | 10,595,066 | 10,506,786 | 11,084,742 | 10,383,535 | 9,553,956 | 8,671,932 | 7,773,679 | 8,103,348 |
| 40-44 | 8,087,950 | 10,464,886 | 10,406,557 | 11,162,910 | 10,096,823 | 9,540,853 | 8,655,887 | 7,753,987 |
| 45-49 | 6,120,986 | 7,979,559 | 10,355,640 | 10,491,250 | 10,619,458 | 10,149,031 | 9,587,136 | 8,697,772 |
| 50-54 | 5,411,600 | 6,031,134 | 7,893,507 | 10,440,362 | 10,435,539 | 10,695,373 | 10,224,243 | 9,660,960 |
| 55-59 | 5,225,557 | 5,307,684 | 5,952,379 | 7,943,495 | 10,007,040 | 10,472,272 | 10,742,157 | 10,275,233 |
| 60-64 | 4,270,022 | 5,075,760 | 5,198,034 | 5,949,383 | 7,584,419 | 9,966,510 | 10,443,327 | 10,727,381 |
| 65-69 | 3,256,822 | 4,067,378 | 4,899,517 | 5,124,090 | 5,759,727 | 7,478,258 | 9,850,110 | 10,342,673 |
| 70-74 | 2,384,157 | 2,972,817 | 3,797,441 | 4,695,126 | 4,826,299 | 5,579,001 | 7,276,801 | 9,619,559 |
| 75-79 | 1,608,161 | 2,001,901 | 2,592,802 | 3,454,623 | 4,215,471 | 4,486,904 | 5,228,380 | 6,872,321 |
| 80-84 | 957,361 | 1,168,898 | 1,529,675 | 2,126,699 | 2,931,359 | 3,581,496 | 3,849,460 | 4,546,896 |
| 85+ | 619,588 | 772,411 | 1,020,158 | 543,184 | 2,376,785 | 3,354,748 | 4,379,637 | 5,178,096 |
|  |  |  |  |  |  |  |  |  |
| **Women** |  |  |  |  |  |  |  |  |
| 0-4 | 8,195,107 | 7,310,954 | 5,762,186 | 5,584,641 | 5,513,460 | 5,472,924 | 5,400,178 | 5,116,327 |
| 5-9 | 7,715,675 | 8,258,275 | 7,391,714 | 5,837,470 | 5,535,031 | 5,475,255 | 5,432,576 | 5,358,423 |
| 10-14 | 8,557,108 | 7,692,086 | 8,233,613 | 7,392,873 | 5,770,130 | 5,491,262 | 5,428,383 | 5,382,837 |
| 15-19 | 9,788,987 | 8,504,561 | 7,657,467 | 8,230,751 | 7,261,200 | 5,736,823 | 5,454,823 | 5,388,520 |
| 20-24 | 10,410,454 | 9,732,932 | 8,470,468 | 7,678,838 | 8,110,515 | 7,303,373 | 5,769,386 | 5,483,833 |
| 25-29 | 11,258,964 | 10,379,373 | 9,709,348 | 8,546,025 | 7,887,894 | 8,231,257 | 7,411,512 | 5,854,440 |
| 30-34 | 10,613,744 | 11,179,802 | 10,321,438 | 9,789,462 | 8,736,594 | 7,839,427 | 8,177,030 | 7,359,723 |
| 35-39 | 10,595,066 | 10,506,786 | 11,084,742 | 10,383,535 | 9,553,956 | 8,671,932 | 7,773,679 | 8,103,348 |
| 40-44 | 8,087,950 | 10,464,886 | 10,406,557 | 11,162,910 | 10,096,823 | 9,540,853 | 8,655,887 | 7,753,987 |
| 45-49 | 6,120,986 | 7,979,559 | 10,355,640 | 10,491,250 | 10,619,458 | 10,149,031 | 9,587,136 | 8,697,772 |
| 50-54 | 5,411,600 | 6,031,134 | 7,893,507 | 10,440,362 | 10,435,539 | 10,695,373 | 10,224,243 | 9,660,960 |
| 55-59 | 5,225,557 | 5,307,684 | 5,952,379 | 7,943,495 | 10,007,040 | 10,472,272 | 10,742,157 | 10,275,233 |
| 60-64 | 4,270,022 | 5,075,760 | 5,198,034 | 5,949,383 | 7,584,419 | 9,966,510 | 10,443,327 | 10,727,381 |
| 65-69 | 3,256,822 | 4,067,378 | 4,899,517 | 5,124,090 | 5,759,727 | 7,478,258 | 9,850,110 | 10,342,673 |
| 70-74 | 2,384,157 | 2,972,817 | 3,797,441 | 4,695,126 | 4,826,299 | 5,579,001 | 7,276,801 | 9,619,559 |
| 75-79 | 1,608,161 | 2,001,901 | 2,592,802 | 3,454,623 | 4,215,471 | 4,486,904 | 5,228,380 | 6,872,321 |
| 80-84 | 957,361 | 1,168,898 | 1,529,675 | 2,126,699 | 2,931,359 | 3,581,496 | 3,849,460 | 4,546,896 |
