# Supplementary material for: Estimated future incidence of malignant mesothelioma in South Korea: Projection from 2014 to 2033
Source: PLoS One. 2017 Aug 17;12(8):e0183404. doi: 10.1371/journal.pone.0183404 (PMC5560642; doi:10.1371/journal.pone.0183404)
Supplement: S2 Table — (DOCX) [file pone.0183404.s002.docx]

**S2 Table. WHO world standard population (2000-2025)**

| **Age Group** | **World standard population** |
| --- | --- |
| 0-4 | 88,569 |
| 5-9 | 86,870 |
| 10-14 | 85,970 |
| 15-19 | 84,670 |
| 20-24 | 82,171 |
| 25-29 | 79,272 |
| 30-34 | 76,073 |
| 35-39 | 71,475 |
| 40-44 | 65,877 |
| 45-49 | 60,379 |
| 50-54 | 53,681 |
| 55-59 | 45,484 |
| 60-64 | 37,187 |
| 65-69 | 29,590 |
| 70-74 | 22,092 |
| 75-79 | 15,195 |
| 80-84 | 9,097 |

| 85+ | 6,348 |
| --- | --- |
| Total | 1,000,000 |
